# Supplementary material for: High-veracity functional imaging in scanning probe microscopy via Graph-Bootstrapping
Source: Nat Commun. 2018 Jun 21;9:2428. doi: 10.1038/s41467-018-04887-1 (PMC6013493; doi:10.1038/s41467-018-04887-1)
Supplement: Supplementary file 1 — Supplementary Information [file 41467_2018_4887_MOESM1_ESM.pdf]

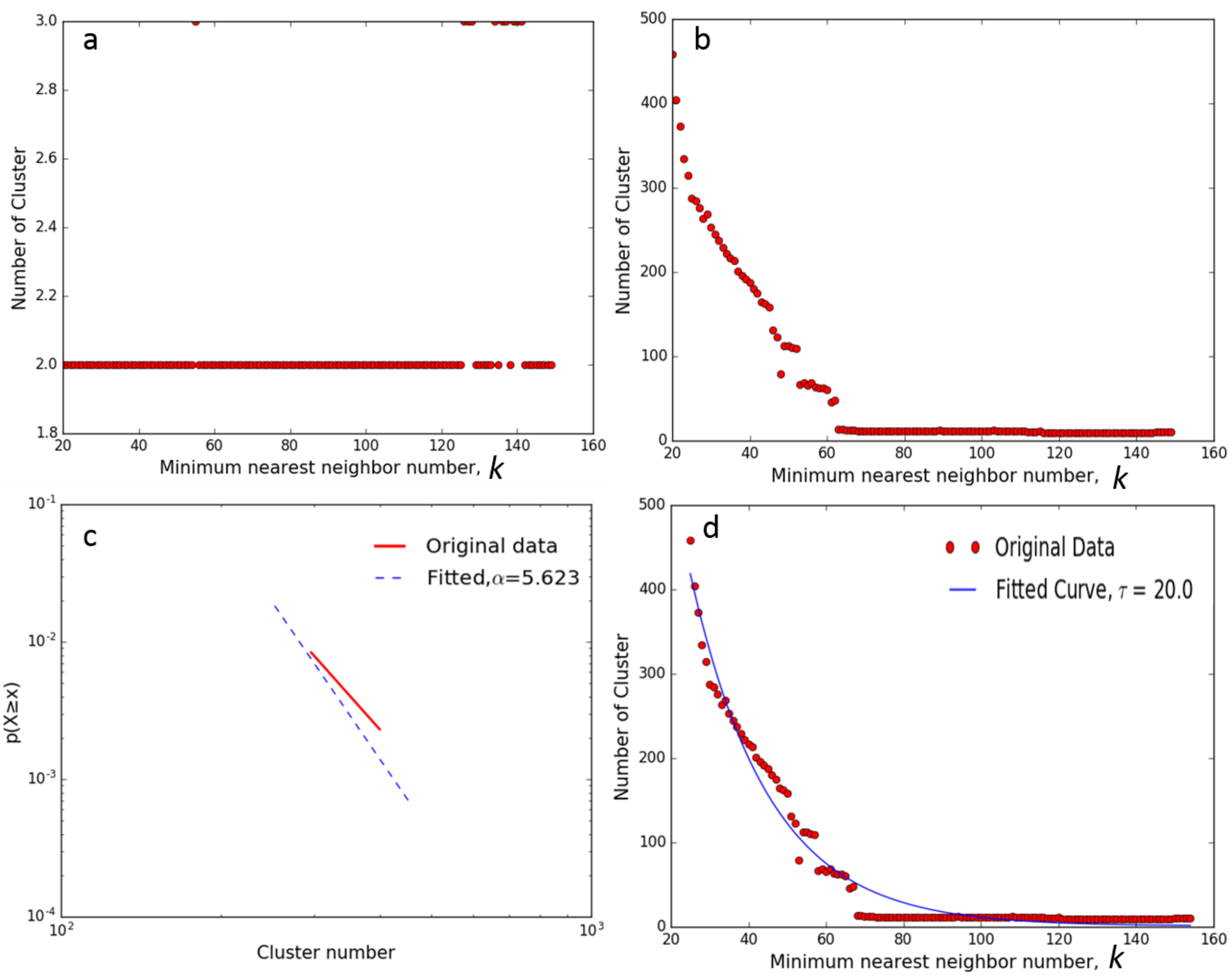

Supplementary Figure 1: Trend of cluster number. (a) Cluster number versus minimum nearest neighbor number, *k*, based on LargeVis manifolds and **(b)** based on Graph-Bootstrapping manifolds; **(c)** Power law fitting of cluster number on Graph-Bootstrapping manifolds and **(d)** Exponential distribution fitting of cluster number trend on Graph-Bootstrapping manifolds.

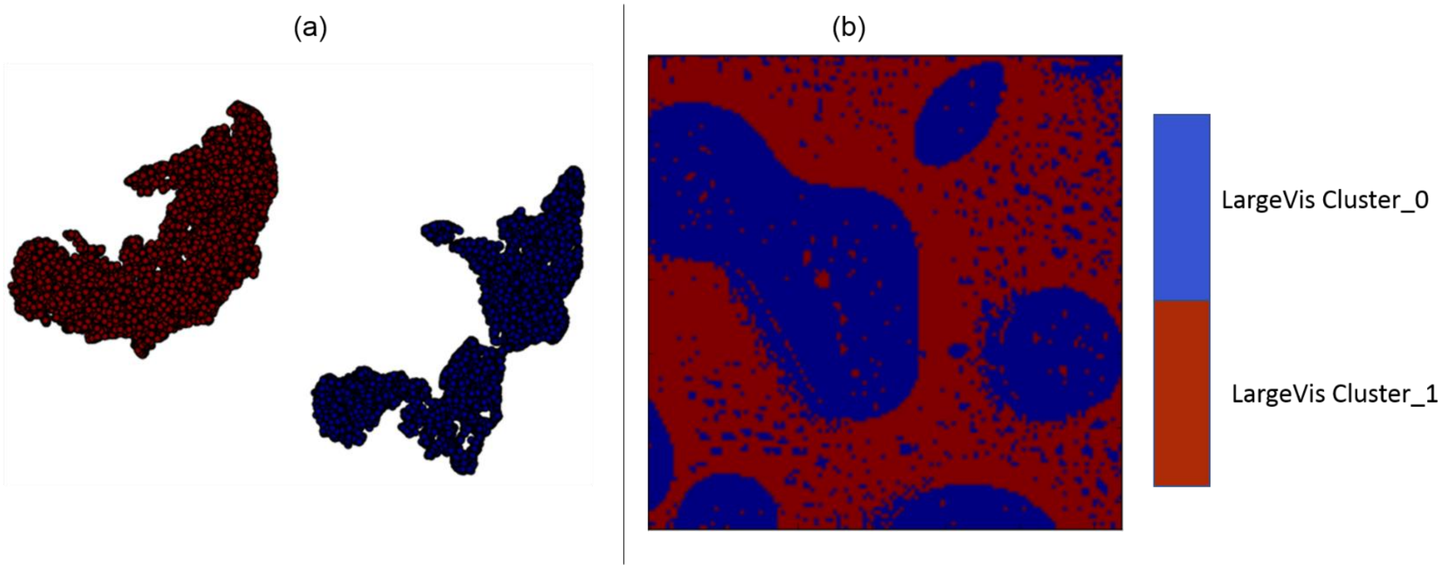

Supplementary Figure 2: LargeVis results based on band excitation scanning probe microscopy datasets collected on the polymer mixture sample. (a) Hierarchical density estimates methods (HDBSCAN) clusters (2 clusters) on LargeVis manifolds and **(b)** Spatial-mappings of the 2 clusters.

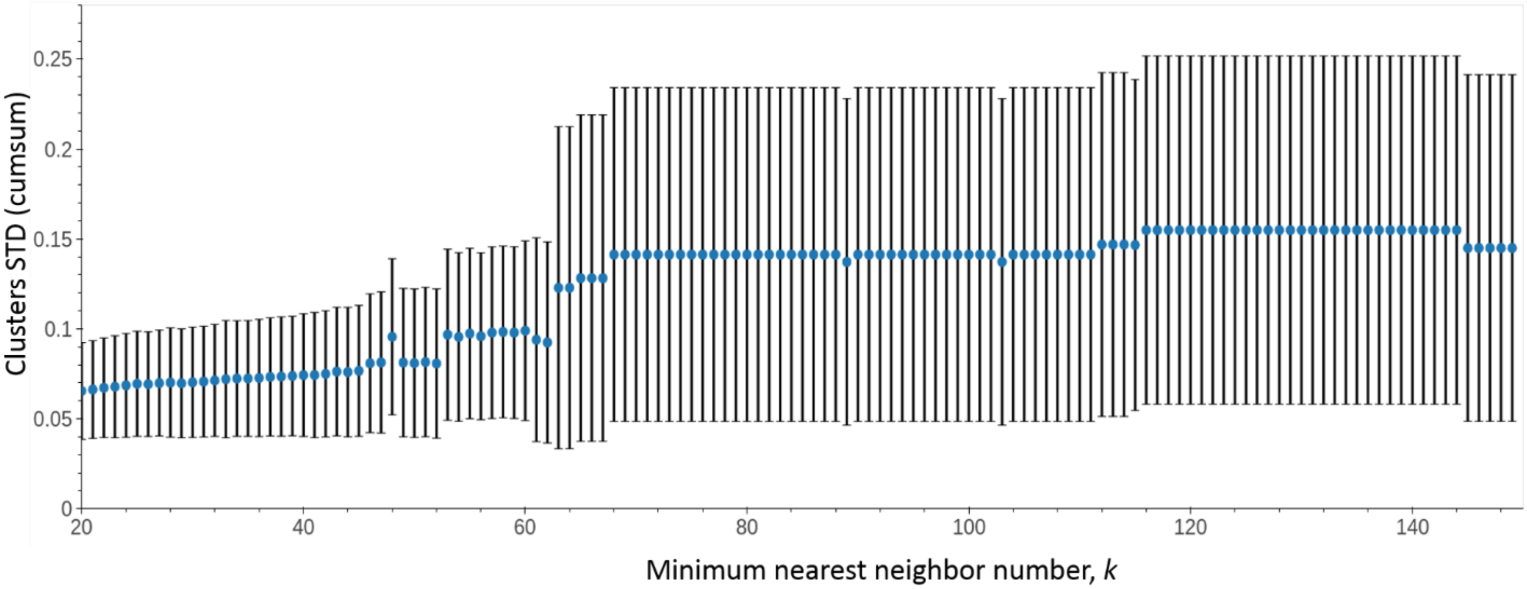

Supplementary Figure 3: Boxplot of clusters' (cumsum) standard deviations (STD) of band excitation scanning probe measurements for different *k* values. The upper and lower bar correspond to the population mean + population std and population mean - population std respectively.

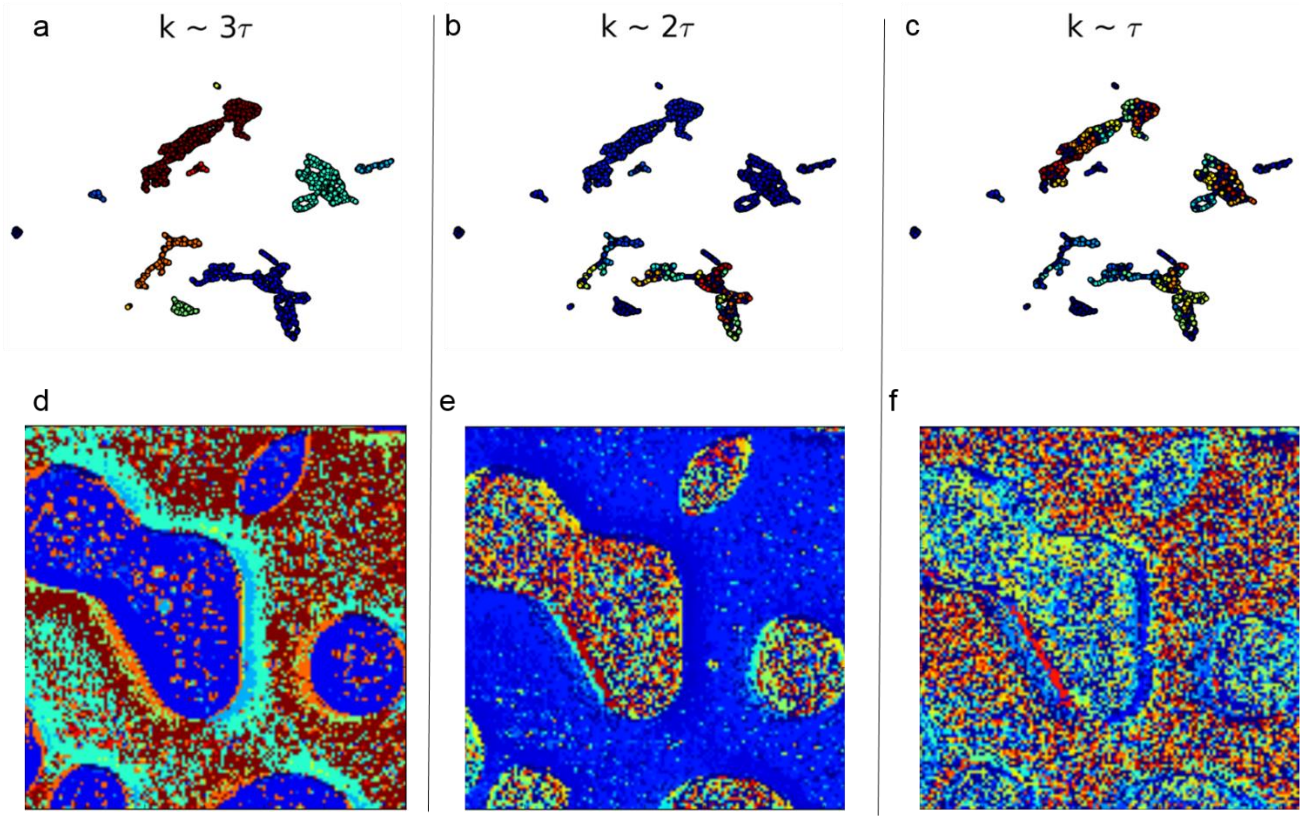

Supplementary Figure 4: Effects on clustering results of Graph-Bootstrapping manifolds by decreasing minimum nearest neighbor number, *k*. For $k \sim 3\tau$, **(a)** is the cluster labels distributed on Graph-Bootstrapping manifold and correspondingly **(d)** is the spatial mappings of cluster labels. For $k \sim 2\tau$, **(b)** is the cluster labels distributed on Graph-Bootstrapping manifold and correspondingly **(e)** is the spatial mappings of cluster labels. For $k \sim \tau$, **(c)** is the cluster labels distributed on Graph-Bootstrapping manifold and correspondingly **(f)** is the spatial mappings of cluster labels.

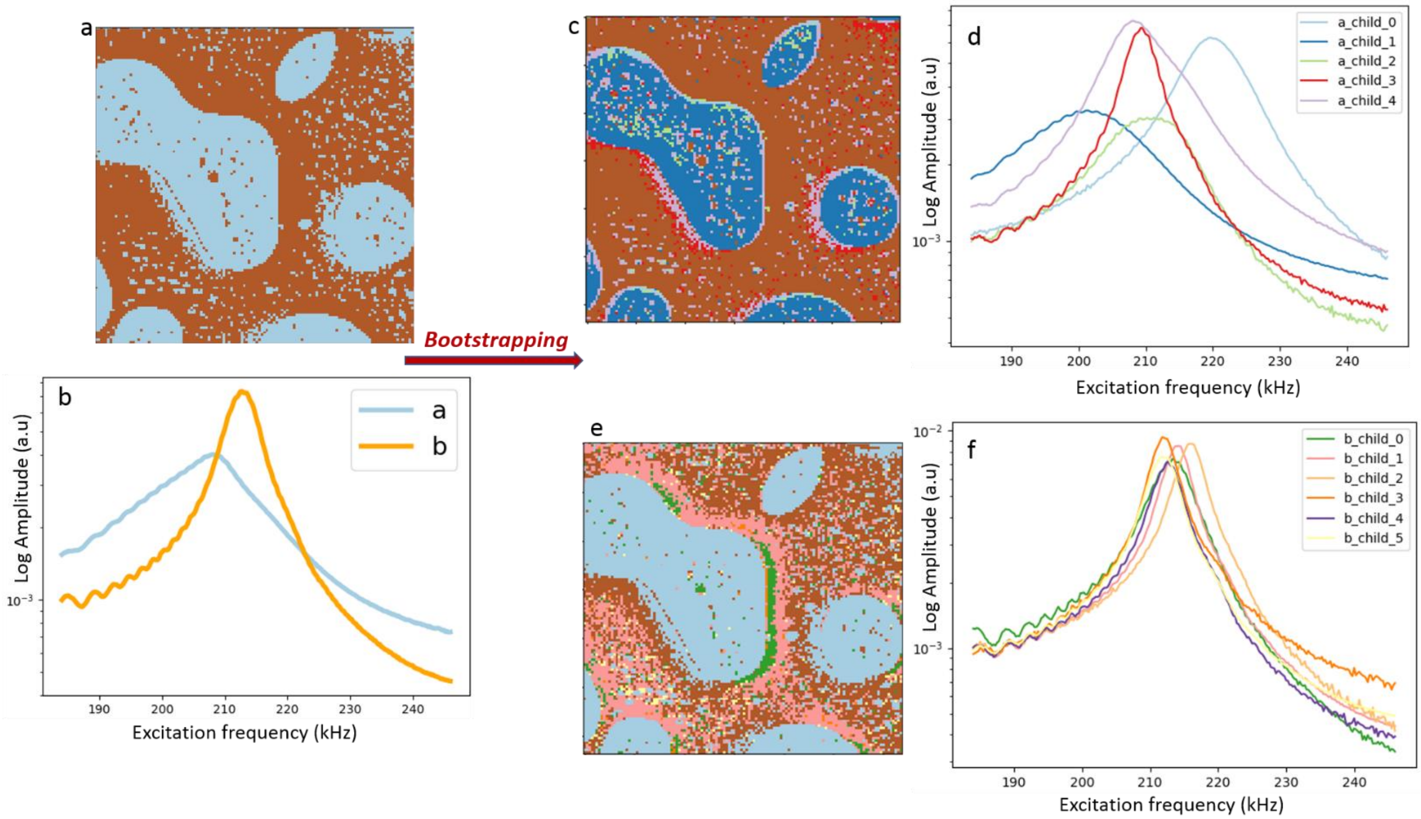

Supplementary Figure 5: Spatial mappings and mean scanning probe microscopy (SPM) signals of LargeVis and Graph-Bootstrapping clusters. (a) Spatial mappings of LargeVis clusters a and b and **(b)** the corresponding mean SPM curves for those two LargeVis clusters. **(c)** The spatial mappings of bootstrapped clusters from LargeVis cluster a and **(d)** the corresponding mean SPM curves. **(e)** The spatial mappings of bootstrapped clusters from LargeVis cluster b and **(f)** the corresponding mean SPM curves.

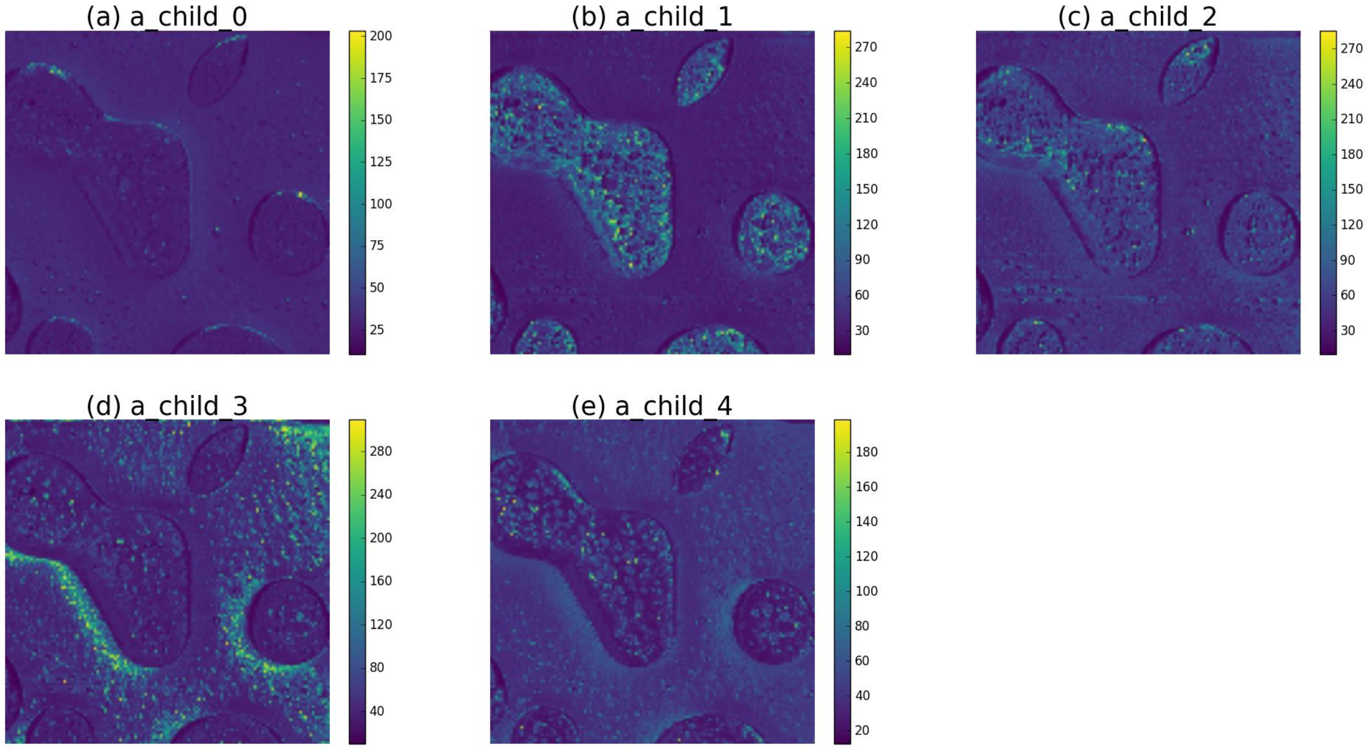

Supplementary Figure 6: Similarity loadings for bootstrapped clusters in Supplementary Fig. 5c,d. Similarity loadings of bootstrapped clusters **(a)** a_child_0, **(b)** a_child_1, **(c)** a_child_2, **(d)** a_child_3 and **(e)** a_child_4 in Supplementary Fig. 5c,d. Similarity loadings are calculated by the inversion of pairwise Euclidean distances between the mean scanning probe microscopy (SPM) curve of the cluster and every raw SPM curve.

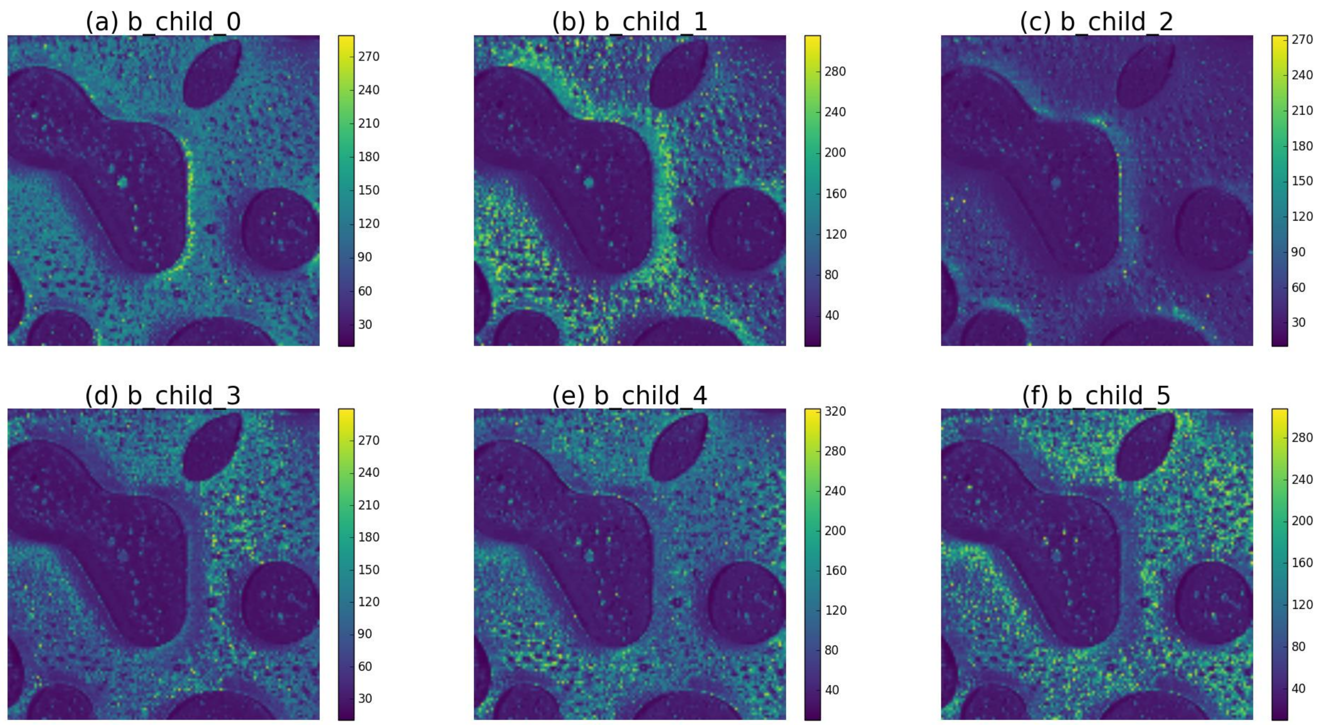

Supplementary Figure 7: Similarity loadings for bootstrapped clusters in Supplementary Fig. 5e,f. Similarity loadings of bootstrapped clusters **(a)** b_child_0, **(b)** b_child_1, **(c)** b_child_2, **(d)** b_child_3, **(e)** b_child_4 and **(f)** b_child_5 in Supplementary Fig. 5e,f. Similarity loadings are calculated by the inversion of pairwise Euclidean distances between the mean scanning probe microscopy (SPM) curve of the cluster and every raw SPM curve.

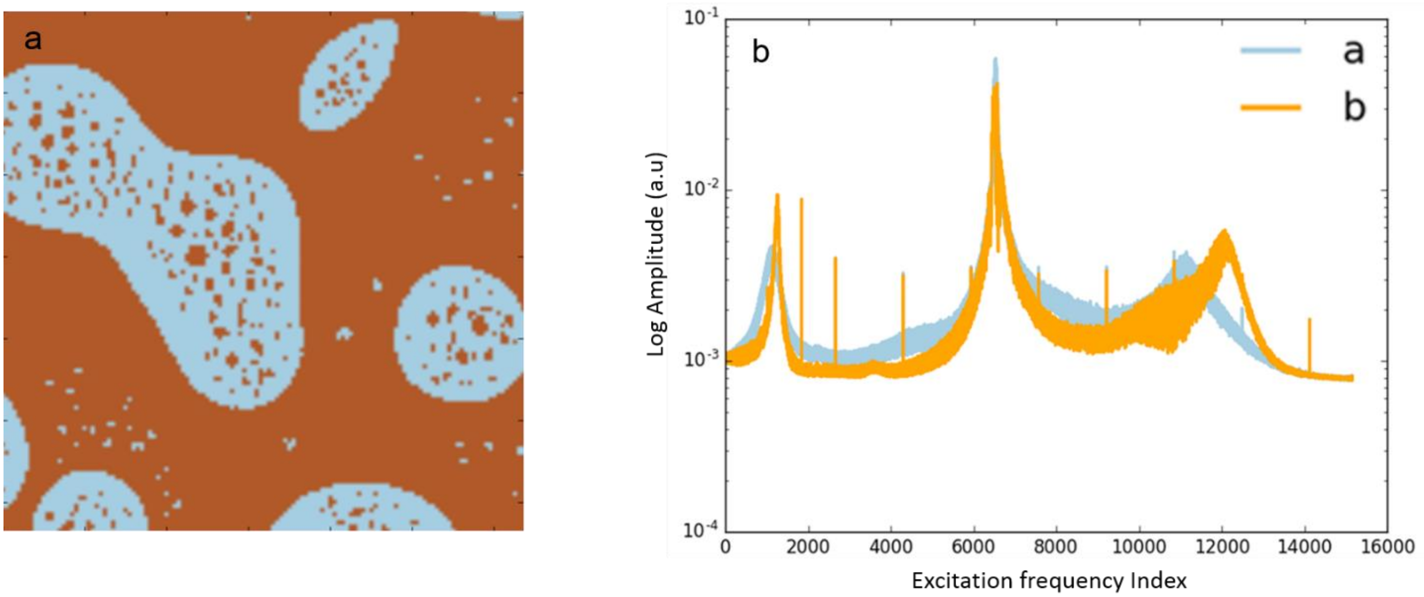

Supplementary Figure 8: Broadband band excitation scanning probe microscopy (Broadband BE-SPM) results. (a) Spatial mappings and **(b)** the mean broadband BE-SPM signals of LargeVis clusters a and b.

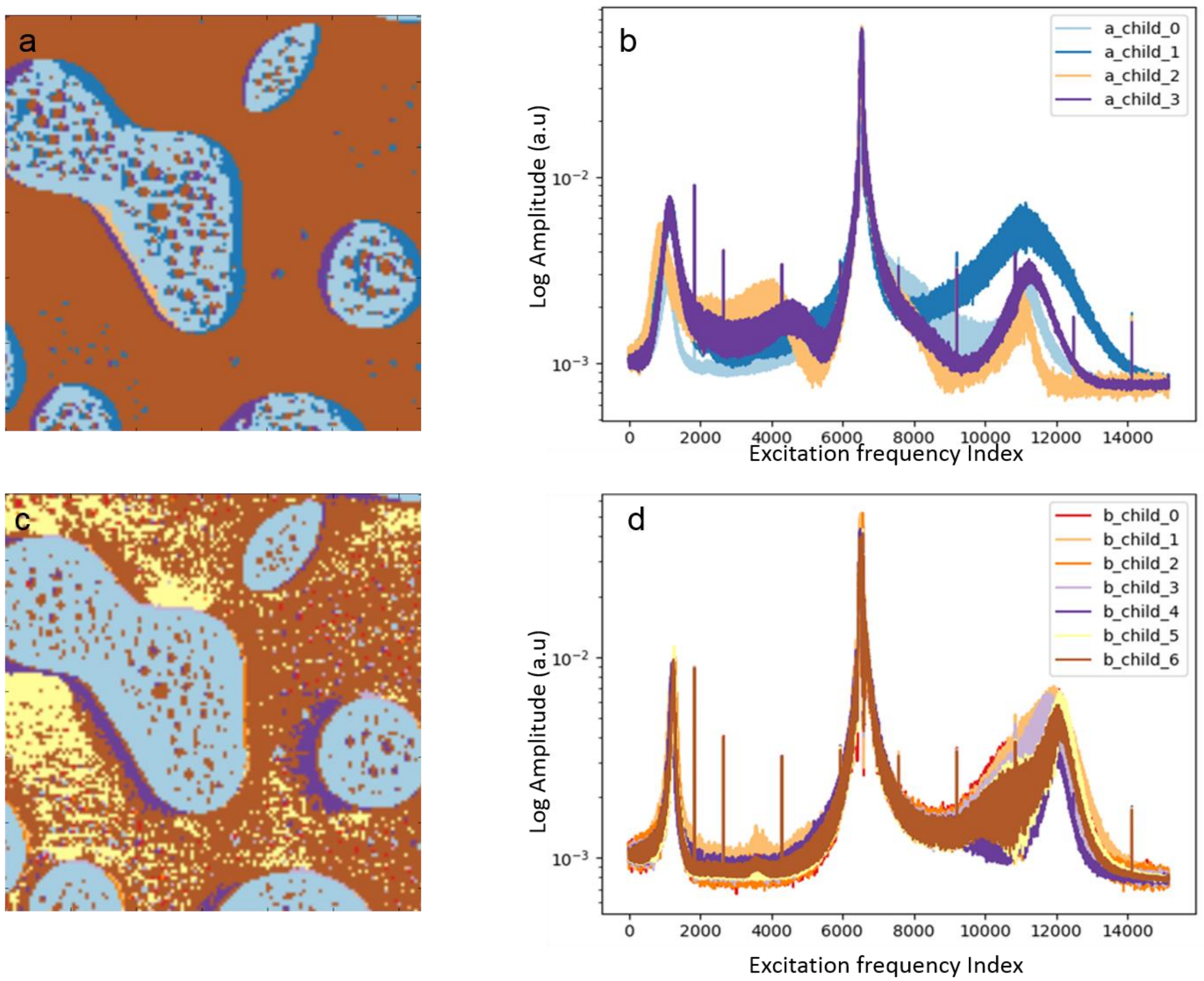

Supplementary Figure 9: Broadband band excitation scanning probe microscopy (Broadband BE-SPM) results. (a) The spatial mappings of bootstrapped clusters from LargeVis cluster a in Supplementary Fig. 8 and **(b)** the corresponding mean broadband BE-SPM curves. **(c)** The spatial mappings of bootstrapped clusters from LargeVis cluster b in Supplementary Fig. 8 and **(d)** the corresponding mean broadband BE-SPM curves.

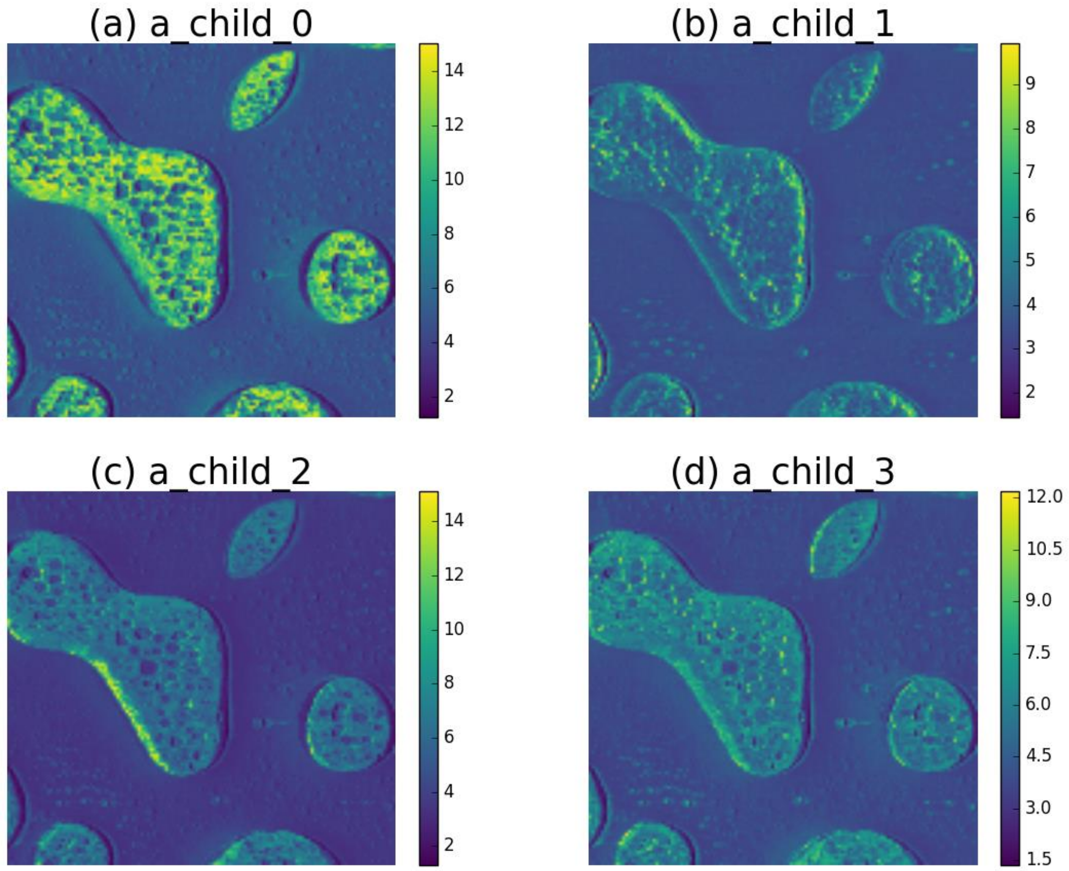

Supplementary Figure 10: Broadband band excitation scanning probe microscopy (Broadband BE-SPM) results. Similarity loadings for bootstrapped clusters in Supplementary Fig. 9a,b. Similarity loadings of bootstrapped clusters **(a)** a_child_0, **(b)** a_child_1, **(c)** a_child_2 and **(d)** a_child_3 in Supplementary Fig. 9a,b. Similarity loadings are calculated by the inversion of pairwise Euclidean distances between the mean broadband BE-SPM curve of the cluster and every raw broadband BE-SPM curve.

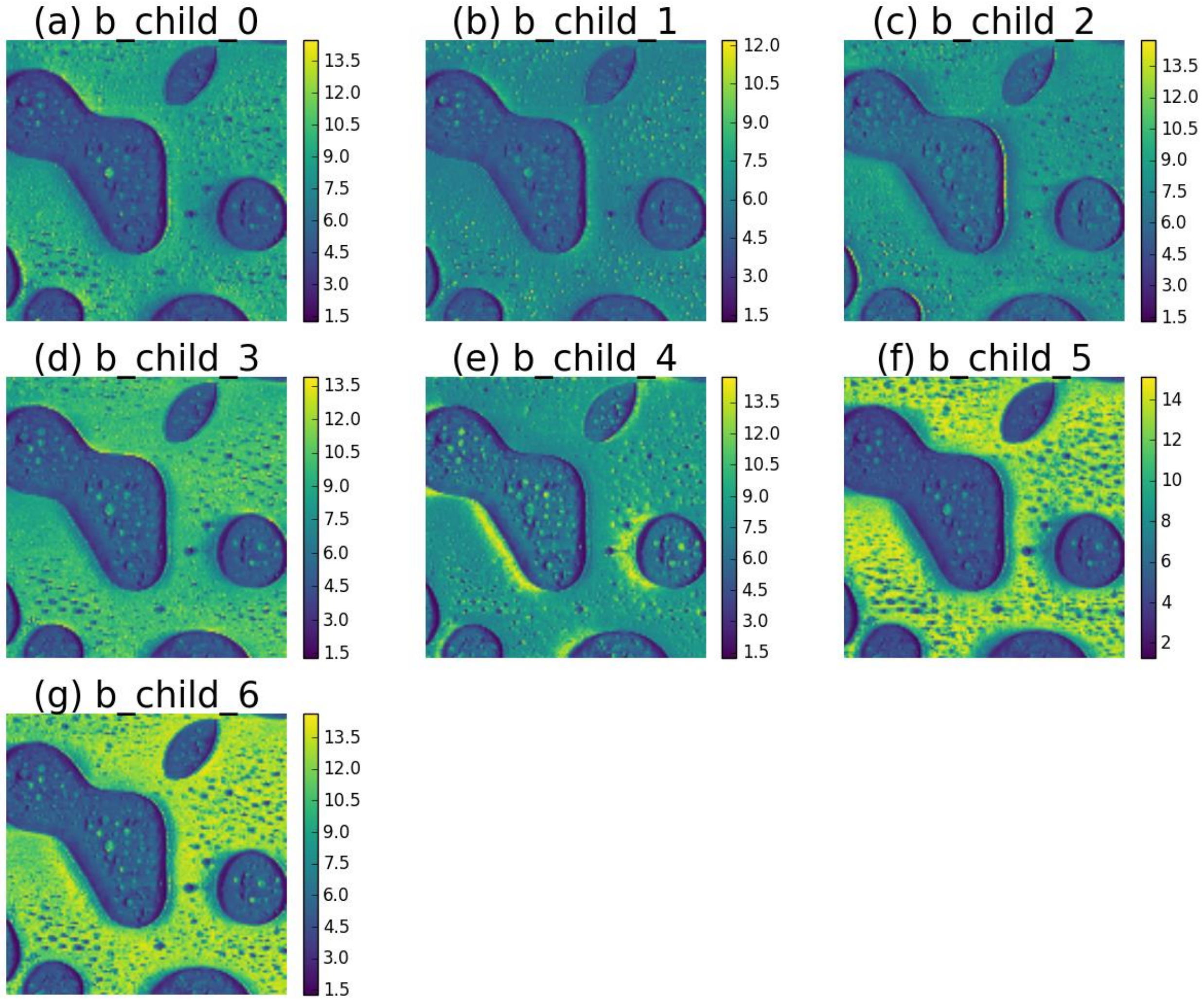

Supplementary Figure 11: Broadband band excitation scanning probe microscopy (Broadband BE-SPM) results. Similarity loadings for bootstrapped clusters in Supplementary Fig. 9c,d. Similarity loadings of bootstrapped clusters **(a)** b_child_0, **(b)** b_child_1, **(c)** b_child_2, **(d)** b_child_3, **(e)** b_child_4, **(f)** b_child_5 and **(g)** b_child_6 in Supplementary Fig. 9c,d. Similarity loadings are calculated by the inversion of pairwise Euclidean distances between the mean broadband BE-SPM curve of the cluster and every raw broadband BE-SPM curve.

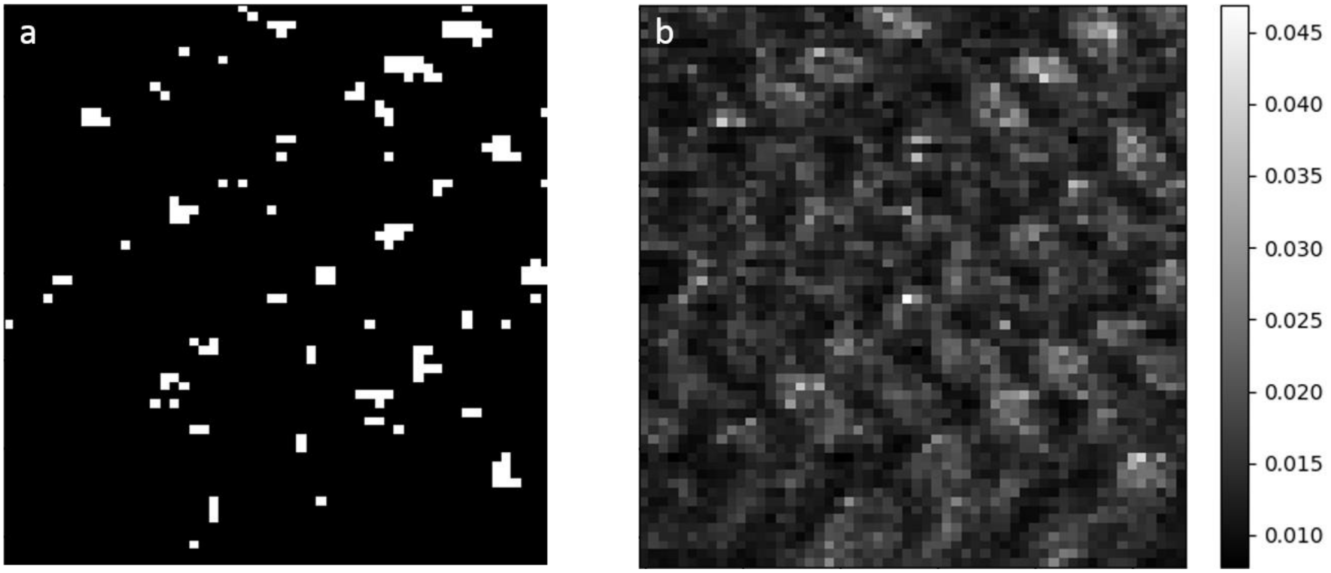

Supplementary Figure 12: Three-dimensional atomic force microscopy (3D-AFM) results. **(a)** Spatial distribution (white) of cluster labels for cluster_0 in Fig. 6 and the corresponding **(b)** Similarity loadings of cluster_0. Similarity loadings are calculated by the inversion of pairwise Euclidean distances between the mean 3D-AFM curve of the cluster_0 (Fig. 6g) and every raw 3D-AFM curve.
